# Metal–Organic Framework-Assisted Synthesis of Compact Fe_2_O_3_ Nanotubes in Co_3_O_4_ Host with Enhanced Lithium Storage Properties

**DOI:** 10.1007/s40820-018-0197-1

**Published:** 2018-04-07

**Authors:** Song Lin Zhang, Bu Yuan Guan, Hao Bin Wu, Xiong Wen David Lou

**Affiliations:** 1 School of Chemical and Biomedical Engineering, Nanyang Technological University, 62 Nanyang Drive, Singapore, 637459 Singapore; 2 School of Materials Science and Engineering, Zhejiang University, Hangzhou, 310027 People’s Republic of China

**Keywords:** Metal–organic framework (MOF), Hierarchical structures, Fe_2_O_3_ nanotubes, Co_3_O_4_, Lithium-ion batteries (LIBs)

## Abstract

Transition metal oxides are promising candidates for the high-capacity anode material in lithium-ion batteries. The electrochemical performance of transition metal oxides can be improved by constructing suitable composite architectures. Herein, we demonstrate a metal–organic framework (MOF)-assisted strategy for the synthesis of a hierarchical hybrid nanostructure composed of Fe_2_O_3_ nanotubes assembled in Co_3_O_4_ host. Starting from MOF composite precursors (Fe-based MOF encapsulated in a Co-based host matrix), a complex structure of Co_3_O_4_ host and engulfed Fe_2_O_3_ nanotubes was prepared by a simple annealing treatment in air. By virtue of their structural and compositional features, these hierarchical composite particles reveal enhanced lithium storage properties when employed as anodes for lithium-ion batteries.

**Electronic supplementary material:**

The online version of this article (10.1007/s40820-018-0197-1) contains supplementary material, which is available to authorized users.

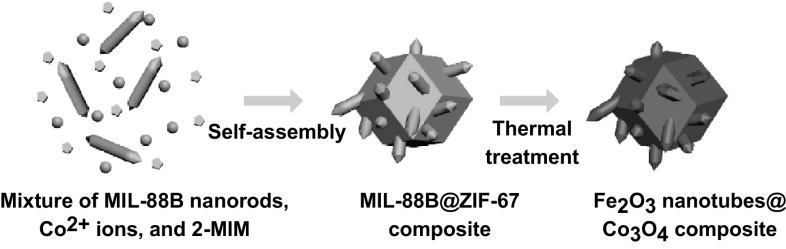

## Highlights

- A metal–organic framework (MOF)-assisted approach is developed for the synthesis of hierarchical composite particles composed of Fe_2_O_3_ nanotubes encapsulated in a Co_3_O_4_ host matrix.
- The hierarchical Fe_2_O_3_ nanotubes@Co_3_O_4_ composite particles exhibit excellent electrochemical performance when evaluated as an anode material for lithium-ion batteries (LIBs).

## Introduction

Lithium-ion batteries (LIBs) have drawn considerable research attention as a rechargeable power source for portable electronic devices and electric vehicles [1, 2]. Until now, graphite has been the most commonly used anode material in commercial LIBs [3]. However, the relatively low theoretical capacity (372 mAh g^−1^) of graphite is inadequate to meet the growing demands of energy density and life span in next-generation batteries [4–7]. Transition metal oxides (TMOs) have been considered as promising electrode materials for LIBs owing to their high specific capacity, low cost, and synthetic versatility to diverse nanostructures [8–11]. As two representative TMOs, iron oxide and cobalt oxide have been actively investigated [12–18]. However, the practical application of these anode materials still faces serious challenges, such as fast capacity fading, poor rate performance caused by large volume changes occurring during the lithiation/delithiation processes, and low intrinsic electric conductivity.

To overcome these drawbacks, diverse approaches have been proposed to improve the lithium storage properties. One effective way is to integrate two or more TMO materials into hybrid nanostructures [3, 19, 20]. The hybrid configuration is expected to retain the advantages of each component and, at the same time, provide synergetic effects that enhance the physicochemical properties such as electrochemical reactivity and mechanical stability [21]. Recently, several iron oxide@cobalt oxide hybrid materials have been reported with enhanced lithium storage capability, such as Fe_2_O_3_@Co_3_O_4_@C composite nanoparticles [22], Fe_2_O_3_@Co_3_O_4_ nanowire arrays [23], and Co_3_O_4_@Fe_2_O_3_ core–shell nanoneedle arrays [24]. In addition, the construction of hierarchical hollow nanostructures was found to be an effective way to accommodate the large volume changes associated with electrochemical reactions [25, 26]. The permeable shells can reduce Li^+^ ion diffusion length and guarantee sufficient electrode–electrolyte contact area. Therefore, a rational design and synthesis approach for iron oxide@cobalt oxide hybrid electrodes with hierarchical hollow nanostructures is expected to yield enhanced lithium storage properties.

In recent years, there have been growing research interest for designing advanced electrode materials with controlled architectures and chemical compositions using metal–organic framework (MOF)-based precursors [27–35]. Most MOF-derived blends are based on simple MOF crystals, and the resulting nanomaterials exhibit relatively simple porous or hollow structures. A rational design of MOF hybrid precursors with novel structures and tailored compositions is highly desirable for the synthesis of high-performance electrode materials [36, 37].

**Fig. 1:**
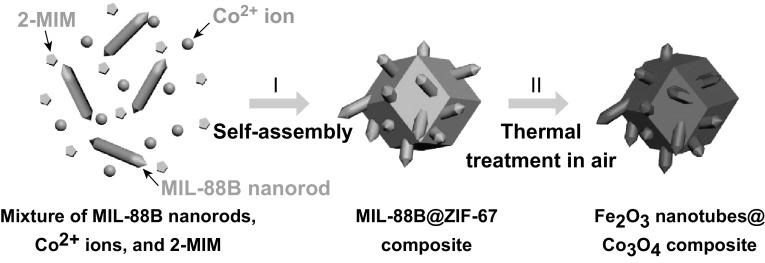
Schematic illustration of the formation process of the Fe_2_O_3_ nanotubes@Co_3_O_4_ composite. (I) Self-assembly of MIL-88B nanorods, Co^2+^ ions, and 2-methylimidazole (2-MIM) to a MIL-88B@ZIF-67 composite. (II) Transformation to Fe_2_O_3_ nanotubes@Co_3_O_4_ composite through thermal treatment in air

In this work, we adopted a MOF-assisted approach for the synthesis of hierarchical composite particles of Fe_2_O_3_ nanotubes encapsulated in Co_3_O_4_ hosts for potential use as an anode material in LIBs. The strategy involves incorporation of MIL-88B (a Fe-based MOF) nanorods in a zeolitic imidazolate framework-67 (ZIF-67, a Co-based MOF) crystal. By a pyrolysis process, the hybrid precursor is transformed into compact Fe_2_O_3_ nanotubes engulfed within the Co_3_O_4_ host matrix (denoted as the Fe_2_O_3_ nanotubesCo_3_O_4_ composites) (Fig. 1). Benefiting from the unique structure and robust matrix, the as-prepared hierarchical Fe_2_O_3_ nanotubes@Co_3_O_4_ composite particles exhibit remarkable electrochemical performance when evaluated as an anode material for LIBs.

## Experimental

### Synthesis of MIL-88B@ZIF-67 Composites

The MIL-88B nanorods were synthesized by following a hydrothermal method reported earlier [38]. In this method, 0.16 g of F127 was first dissolved in 15 mL of deionized water to which 0.179 g of FeCl_3_·6H_2_O was added. The solution mixture was stirred for 1 h, and 0.6 mL of acetic acid was added to it. After stirring for 1 h, 0.06 g of 2-aminoterephthalic acid was injected. It was stirred for another 2 h, after which the reaction mixture was transferred into an autoclave and crystallized for 24 h at 110 °C. The resulting product was washed with ethanol several times. It was then dispersed with 10 mL of methanol solution containing 0.5 g of polyvinylpyrrolidone (PVP, *M*_w_ = 40,000), and the mixture was stirred at room temperature for 12 h. The PVP-functionalized MIL-88B nanorods were collected by centrifugation, washed several times with methanol, and dispersed in 15 mL of methanol for further use. To synthesize the MIL-88B@ZIF-67 composite, 0.8 mL of the MIL-88B nanorod suspension, 5 mL of 80 mM 2-methylimidazole (2-MIM) solution, and 3 mL of 20 mM Co(NO_3_)_2_·6H_2_O solution were mixed and allowed to react at room temperature for 4 h without stirring. The reaction product was extracted by centrifugation, washed with methanol several times, and vacuum-dried overnight.

### Thermal Synthesis of Fe_2_O_3_ Nanotubes@Co_3_O_4_ Composites

The as-formed MIL-88B@ZIF-67 composite was placed in a ceramic boat and heated to 500 °C at a ramp rate of 5 °C min^−1^ in a tube furnace under ambient atmosphere. The temperature was maintained for 2 h after which the furnace was naturally cooled to room temperature.

### Materials Characterization

Field-emission scanning electron microscope (FESEM; JEOL-6700F) and transmission electron microscope (TEM; JEOL-2010) were used to examine the morphology and structure of the prepared samples. The composition was analyzed by an energy-dispersive X-ray analysis (EDX) equipment attached to the FESEM instrument. The crystal phase was examined using a Bruker D2 Phaser X-ray diffractometer. Elemental mapping and high-angle annular dark-field scanning transmission electron microscopy (HAADF-STEM) were performed in a JEOL-2100F electron microscope. Nitrogen sorption isotherms were measured using Autosorb 6B.

### Electrochemical Measurements

Electrochemical measurements were carried out using CR2032 coin-type half cells. The working electrode consists of an active material (here, Fe_2_O_3_ nanotubes@Co_3_O_4_ composite particles), carbon black (Super-P–Li), and a polymer binder (polyvinylidene fluoride) in the weight ratio of 70:20:10. The loading mass of the active material is approximately 0.5–0.8 mg cm^−2^ for each electrode. Lithium foil was used for both the counter and reference electrodes. LiPF_6_ (1.0 M) in a 50:50 (w/w) mixture of ethylene carbonate and diethyl carbonate was used as the electrolyte. The cell assembly was placed in an Ar-filled glove box with moisture and oxygen concentrations below 1.0 ppm. The galvanostatic charge–discharge tests were performed with a Neware battery test system.

## Results and Discussion

**Fig. 2:**
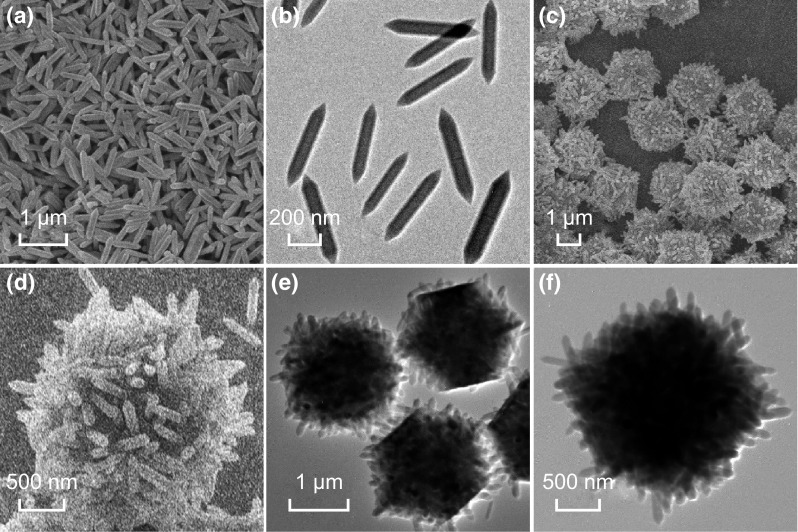
FESEM images of **a** PVP-functionalized MIL-88B nanorods and **c, d** MIL-88B@ZIF-67 composites. TEM images of **b** PVP-functionalized MIL-88B nanorods and **e, f** MIL-88B@ZIF-67 composites

MOF-based precursors have been widely used to fabricate inorganic functional materials with various micro-/nanostructures. To enable the synthesis of complex micro-/nanostructured materials, composite MOF precursors with multiple components are highly desirable, even though the synthesis is quite challenging [39, 40]. A facile solution-based method developed here easily facilitates the assembly of pre-synthesized MIL-88B nanorods within each ZIF-67 crystal. The MIL-88B nanorods synthesized through a modified hydrothermal method [38] are firstly functionalized with PVP on their surface (Fig. 2a, b). The incorporation of MIL-88B nanorods in the ZIF-67 crystal host was carried out by mixing MIL-88B nanorods with the metal ions and organic ligands of ZIF-67 in methanol, and maintaining at room temperature for 4 h [41]. FESEM images show the morphology of the resulting composite particles (Fig. 2c, d). The uniform particles with a size of 2–3 μm exhibit a very rough surface composed of randomly oriented MIL-88B nanorods. TEM images further reveal the solid feature of each ZIF-67 crystal, with numerous MIL-88B nanorods uniformly distributed within each particle (Fig. 2e, f).

**Fig. 3:**
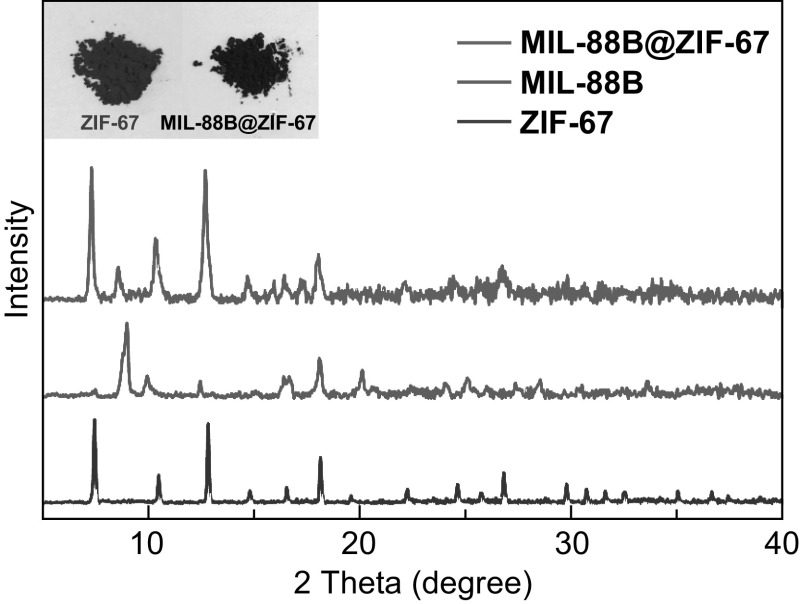
XRD patterns of MIL-88B@ZIF-67 composites, MIL-88B and ZIF-67. Insets show the digital photographs of the MIL-88B@ZIF-67 composites and ZIF-67

As seen in the XRD patterns (Fig. 3), the MIL-88B@ZIF-67 composites exhibit the diffraction peaks of both MIL-88B and ZIF-67 with high crystallinity. In addition, the successful incorporation of MIL-88B nanorods in ZIF-67 crystals can be visualized by the dark red color of the resultant product, which is quite different from the purple color of pristine ZIF-67 (insets of Fig. 3).

**Fig. 4:**
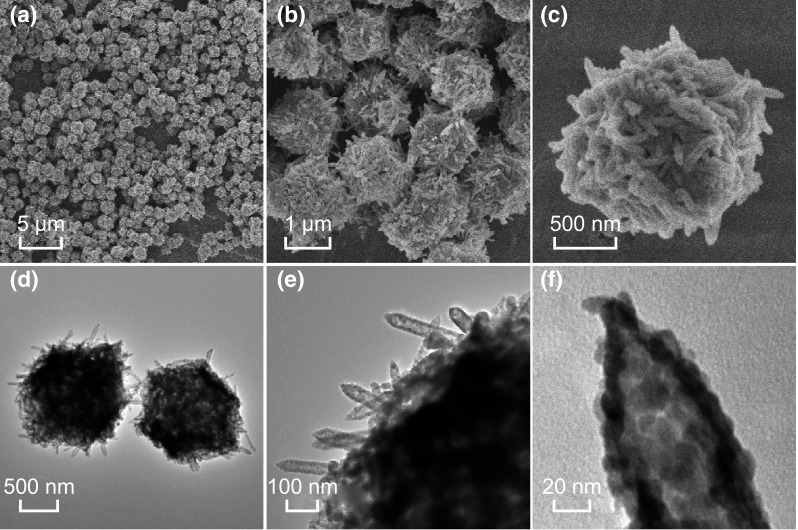
**a**–**c** FESEM images and **d–f** TEM images of the Fe_2_O_3_ nanotubes@Co_3_O_4_ composites

The Fe_2_O_3_ nanotubes@Co_3_O_4_ composites were synthesized by thermal treatment of MIL-88B@ZIF-67 precursors at 500 °C in air. Figure 4a shows a low-magnification FESEM image of the as-derived Fe_2_O_3_ nanotubes@Co_3_O_4_ composite particles. The composite sample preserves the morphology of its MOF precursor even after annealing treatment. A shrinkage of both rhombic dodecahedral hosts and rod-shaped guests is observed after the pyrolysis process, while the surface roughness of the annealed particles increased (Fig. 4b, c). The structure of the as-derived Fe_2_O_3_ nanotubes@Co_3_O_4_ composite was further examined by TEM. As shown in Fig. 4d, e, the Fe_2_O_3_ nanotubes derived from MIL-88B nanorods are evenly distributed in the Co_3_O_4_ host matrix. The length and diameter of the Fe_2_O_3_ nanotubes are about 455 and 55 nm, respectively (Fig. S1). A closer observation of the edge of a Fe_2_O_3_ nanotubes@Co_3_O_4_ composite particle reveals that each Fe_2_O_3_ nanotube is composed of small nanocrystallites (Fig. 4f). High-resolution TEM images (Fig. S2) confirm this observation, in which the lattice fringes assigned to the crystal planes of Fe_2_O_3_ and Co_3_O_4_ are clearly discernible.

**Fig. 5:**
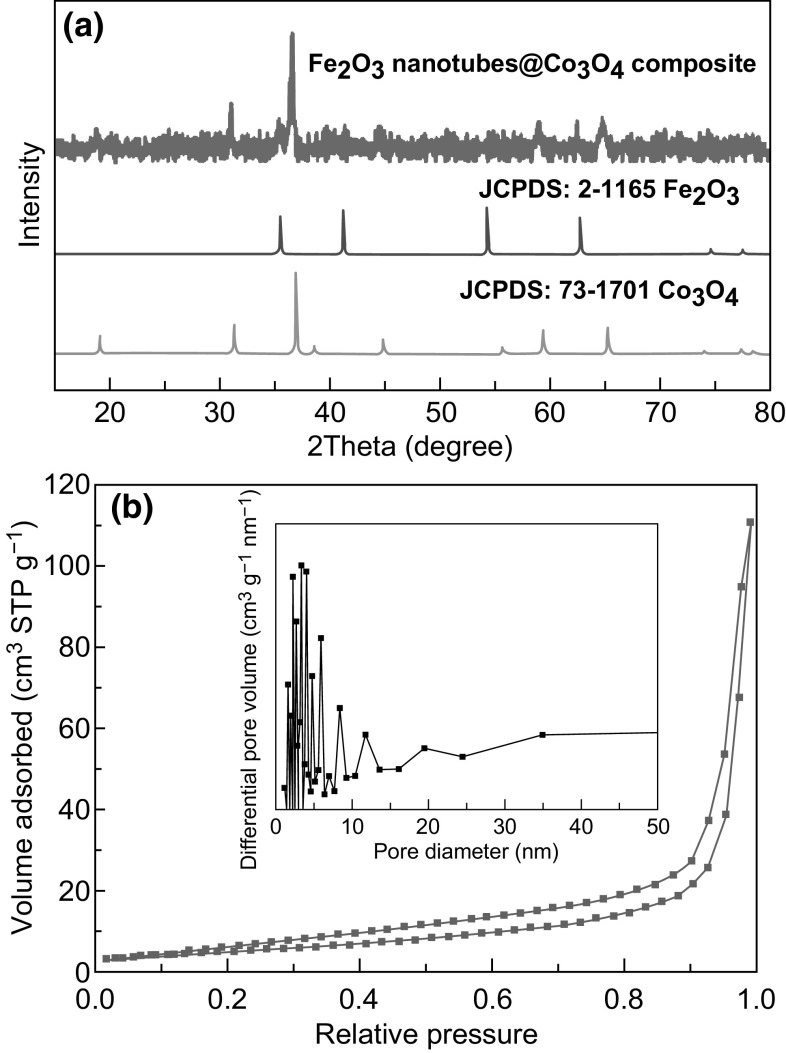
**a** XRD pattern of the Fe_2_O_3_ nanotubes@Co_3_O_4_ composites. **b** N_2_ sorption isotherms of the Fe_2_O_3_ nanotubes@Co_3_O_4_ composites. Inset gives the pore size distribution

**Fig. 6:**
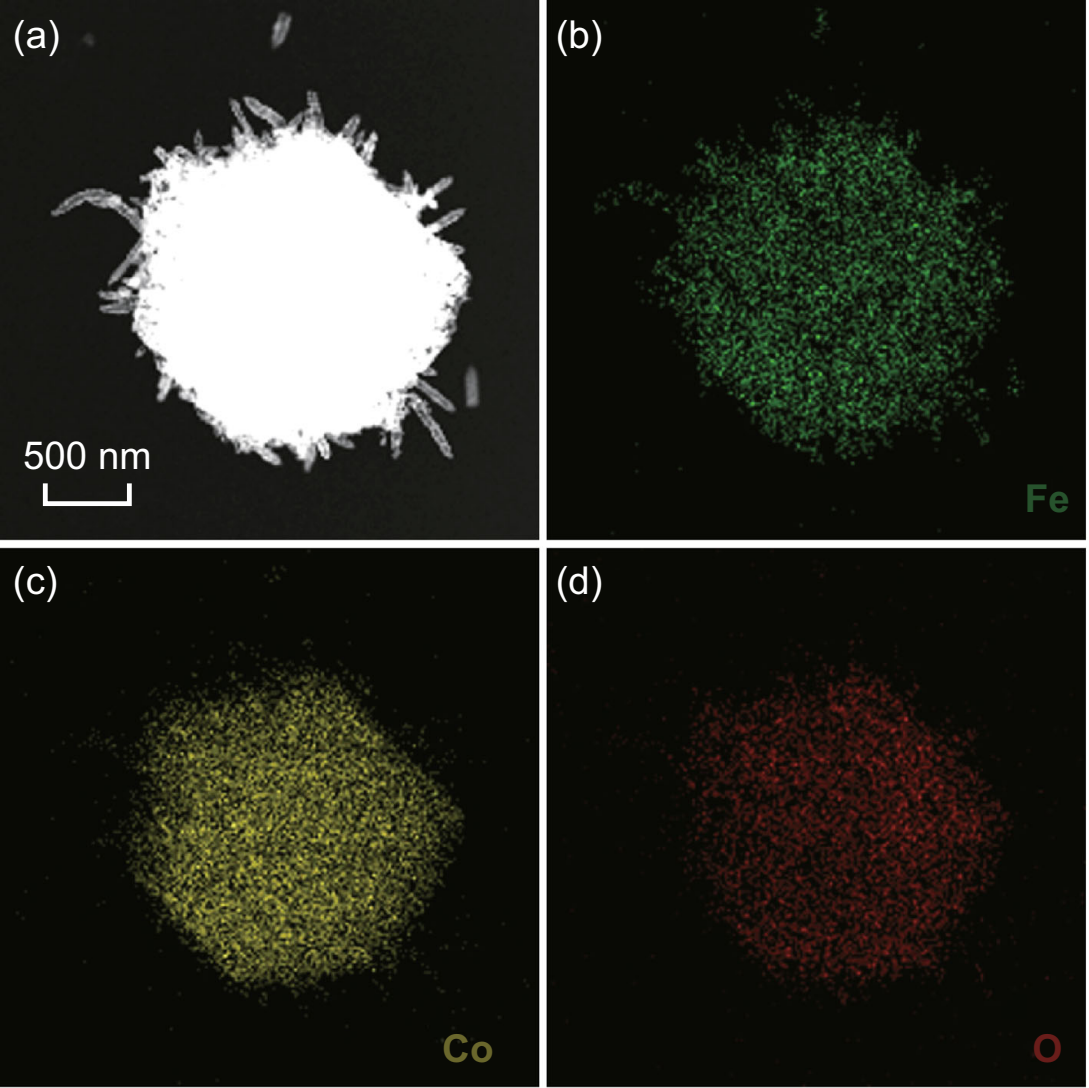
**a** HAADF-STEM image of Fe_2_O_3_ nanotubes@Co_3_O_4_ composite particles. Elemental mapping images of **b** Fe, **c** Co, and **d** O

The crystalline phases in the composite material were confirmed by powder XRD analysis (Fig. 5a). The XRD patterns were indexed to a mixture of δ-Fe_2_O_3_ phase (JCPDS card No. 2-1165) and cubic Co_3_O_4_ phase (JCPDS card No. 73-1701). The nitrogen sorption measurement indicates a moderate surface area of ~ 18 m^2^ g^−1^ for the Fe_2_O_3_ nanotubes@Co_3_O_4_ composite (Fig. 5b). Such a compact architecture may help to provide relatively good structural robustness and suppress parasitic side reactions between electrode and electrolyte [42, 43]. For comparison, Fe_2_O_3_ and Co_3_O_4_ nanostructures (derived from MIL-88B and ZIF-67) reveal Brunauer–Emmett–Teller (BET) surface areas of 7 and 45 m^2^ g^−1^, respectively (Fig. S3). From an analysis of the chemical composition (by EDX), the Fe to Co molar ratio was obtained as 0.58:1 (Fig. S4). This value is very close to the experimental molar ratio (0.59:1) of Fe to Co that was used for synthesis. The spatial distribution of iron and cobalt oxides is shown in Fig. 6, as obtained from HAADF-STEM images and elemental mapping. The Fe_2_O_3_ nanotubes are seen to be evenly dispersed in the Co_3_O_4_ host matrix. X-ray photoelectron spectroscopy (XPS) measurements helped to identify the various valence states of Fe, Co, and O in the Fe_2_O_3_ nanotubes@Co_3_O_4_ composites (Fig. S5a). The binding energies of Fe 2p_3/2_ and 2p_1/2_ peaks are located at 707.8 and 721.3 eV, respectively, confirming the presence of Fe^3+^ state in Fe_2_O_3_ (Fig. S5b). The binding energies at 776.7 and 792.1 eV in the Co 2p spectrum are attributed to the Co^2+^ and Co^3+^ states in Co_3_O_4_ (Fig. S5c). The O 1 s spectrum can be deconvoluted into two bands at 284.4 and 285.1 eV, which are assigned to the O^2−^ state in Fe_2_O_3_ and Co_3_O_4_, respectively (Fig. S5d).

**Fig. 7:**
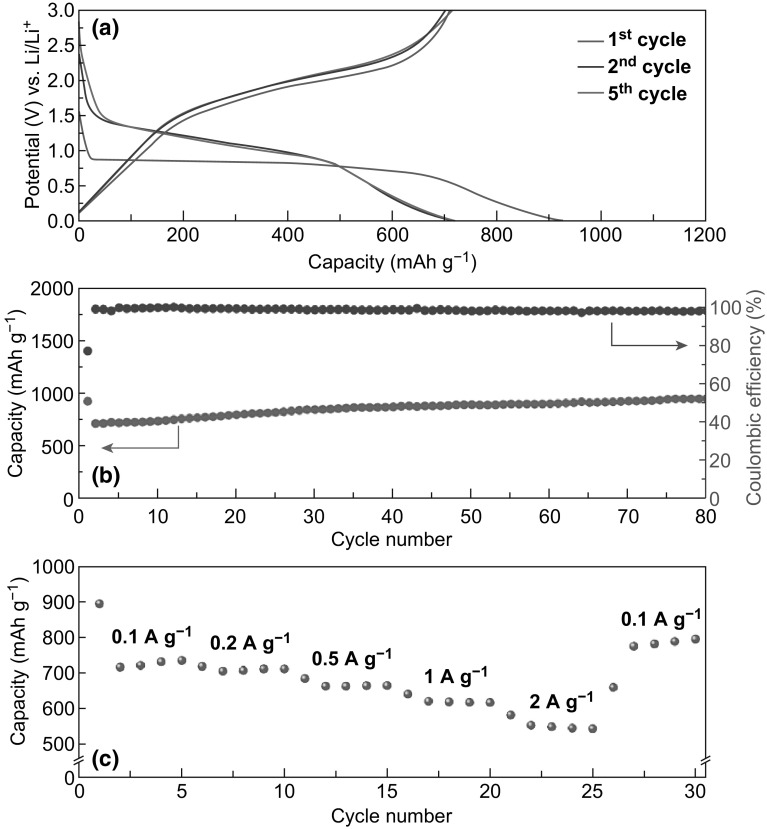
Electrochemical characterization of the Fe_2_O_3_ nanotubes@Co_3_O_4_ composite as an anode material in LIBs. **a** Discharge–charge voltage profiles in the voltage range 0.01–3.0 V at a current density of 0.5 A g^−1^. **b** Cycling performance and corresponding coulombic efficiency at a current density of 0.5 A g^−1^. **c** Rate performance at various current densities from 0.1 to 2 A g^−1^

Subsequently, we evaluated the electrochemical lithium storage properties of Fe_2_O_3_ nanotubes@Co_3_O_4_ composite particles for use as an anode material in LIBs. Figure 7a shows the representative discharge–charge voltage profiles of Fe_2_O_3_ nanotubes@Co_3_O_4_ composite particles at a current density of 0.5 A g^−1^ within a cutoff voltage window of 0.01–3.0 V. The initial discharge and charge capacities are 921.9 and 709.8 mAh g^−1^, respectively, with a high initial coulombic efficiency of 77.0%. The long discharge plateau at 0.84 V during the first cycle corresponds to the insertion of Li^+^ ions into Fe_2_O_3_/Co_3_O_4_, complete reduction of Fe_2_O_3_/Co_3_O_4_ to metallic Fe/Co, and solid electrolyte interphase (SEI) film formation [22–24]. After the first cycle, the capacity becomes stable. Figure 7b shows the cycling performance of the Fe_2_O_3_ nanotubes@Co_3_O_4_ composite particles at a current density of 0.5 A g^−1^. The capacity decays from the initial value of 922 to 710 mAh g^−1^ in the second cycle. Afterward, the capacity gradually increases to 951 mAh g^−1^ at the end of 80 cycles. The increase in capacity during cycling is commonly observed in many metal oxide-based anode materials [44, 45]. This phenomenon might be associated with the gradual activation of the Fe_2_O_3_@Co_3_O_4_ composite during cycling, which enhances the accessibility of lithium ions in the electrode material [46, 47]. The FESEM and TEM images of the Fe_2_O_3_ nanotubes@Co_3_O_4_ electrode after cycling are shown in Fig. S6. It is seen that the Fe_2_O_3_ nanotubes@Co_3_O_4_ composites retain their hierarchical structure even after 80 cycles. Few tubular subunits can still be observed on the edge of the hybrid particle. The cycling performance at a higher current density of 1.0 A g^−1^ is further testimony to the electrochemical stability of the hierarchical structure during the lithiation/delithiation processes (Fig. S7). For comparison, MIL-88B- and ZIF-67-derived Fe_2_O_3_ and Co_3_O_4_ nanostructures were also prepared by pyrolysis of the corresponding MOF precursors and studied. These exhibited inferior electrochemical stability (Fig. S8). Further, as shown in Fig. 7c, the Fe_2_O_3_ nanotubes@Co_3_O_4_ composite particles display good rate capability at discharge–charge current rates ranging from 0.1 to 2 A g^−1^. The average specific capacities observed are 731, 717, 699, 628, and 554 mAh g^−1^ at current densities of 0.1, 0.2, 0.5, 1, and 2 A g^−1^, respectively. After the high-rate discharge/charge cycling, a high specific capacity of 791 mAh g^−1^ was seen to be retained even when the current density returned to a low value of 0.1 A g^−1^. To further investigate the mechanism of lithium storage in Fe_2_O_3_ nanotubes@Co_3_O_4_ composites, the cyclic voltammetry (CV) behavior of various cycles was investigated (Fig. S9). In the first cycle, cathodic peaks were observed at 1.72 and 0.40 V that correspond to the insertion of Li^+^ into Fe_2_O_3_/Co_3_O_4_ and complete reduction of Fe_2_O_3_/Co_3_O_4_ to metallic Fe/Co, respectively [23]. The peaks at 1.70 and 2.13 V are ascribed to the delithiation processes and restoration of Fe_2_O_3_/Co_3_O_4_ [22, 24]. The subsequent curves show good reproducibility, with two cathodic peaks at 0.69 and 1.30 V and two anodic peaks at 1.70 and 2.13 V. The conversion reaction of Fe_2_O_3_/Co_3_O_4_ with Li^+^ is schematically illustrated (Fig. S10) to show the formation of metallic Fe/Co nanoparticles embedded in a matrix of Li_2_O. These results demonstrate that the Fe_2_O_3_ nanotubes@Co_3_O_4_ composite particles possess excellent electrochemical kinetics and lithium storage characteristics comparable to many other Fe_2_O_3_, Co_3_O_4_, and their composites, which are useful for electrode materials, as reported previously (Table S1).

Overall, we regard that the outstanding lithium storage properties are attributable to a combination of the following factors. First, the assembly of compact Fe_2_O_3_ nanotubes in each Co_3_O_4_ host provides synergistic effects between two metal oxides with slightly different redox potentials [22–24]. This facilitates the electrochemical reactions and guarantees high energy density. Second, the hierarchical multilevel cavities and robust architecture lead to an increase in the electrode/electrolyte contact area and help to accommodate the strain of Li^+^ insertion/extraction, hence contributing to good cycling stability. Finally, the nanosized subunits facilitate electronic/Li^+^ transport in the electrode material, ensuring enhanced electrochemical activity. All of the above make the Fe_2_O_3_ nanotubes@Co_3_O_4_ composite particles a highly promising anode material for LIBs.

## Conclusion

A novel MOF-assisted strategy has been developed to construct a complex hierarchical nanostructure consisting of compact Fe_2_O_3_ nanotubes encapsulated in Co_3_O_4_ host. The synthesis involves incorporation of MIL-88B nanorods in the ZIF-67 polyhedron host followed by a thermal treatment process in air to convert the MIL-88B nanorods and ZIF-67 polyhedron to Fe_2_O_3_ nanotubes and Co_3_O_4_ host, respectively. Benefiting from the unique structural and compositional advantages, the as-prepared hierarchical Fe_2_O_3_ nanotubes@Co_3_O_4_ composite exhibits outstanding electrochemical properties with good rate capability and excellent cycling stability as an anode material for LIBs. Our study sheds new light on the controlled synthesis of complex hollow structures for various energy-related applications.

## Electronic supplementary material

Below is the link to the electronic supplementary material.

Supplementary material 1 (PDF 692 kb)
