# Supplementary material for: Metal–Organic Framework-Assisted Synthesis of Compact Fe2O3 Nanotubes in Co3O4 Host with Enhanced Lithium Storage Properties
Source: Nanomicro Lett. 2018 Apr 7;10(3):44. doi: 10.1007/s40820-018-0197-1 (PMC6199090; doi:10.1007/s40820-018-0197-1)
Supplement: Supplementary file 1 — Supplementary material 1 (PDF 692 kb) [file 40820_2018_197_MOESM1_ESM.pdf]

Supporting Information for

Metal-Organic Framework-Assisted Synthesis of Compact Fe_2O_3 Nanotubes in Co_3O_4 Host with Enhanced Lithium Storage Properties

Songlin Zhang[1], Buyuan Guan[1,*], Haobin Wu[2], Xiongwen (David) Lou[1,*]

[1]School of Chemical and Biomedical Engineering, Nanyang Technological University, 62 Nanyang Drive, Singapore 637459, Singapore

[2]School of Materials Science and Engineering, Zhejiang University, Hangzhou 310027, People's Republic of China

*Corresponding authors. E-mail: xwlou@ntu.edu.sg (Xiongwen (David) Lou); guanbuyuan@ntu.edu.sg (Buyuan Guan)

Supplementary Figures and Tables

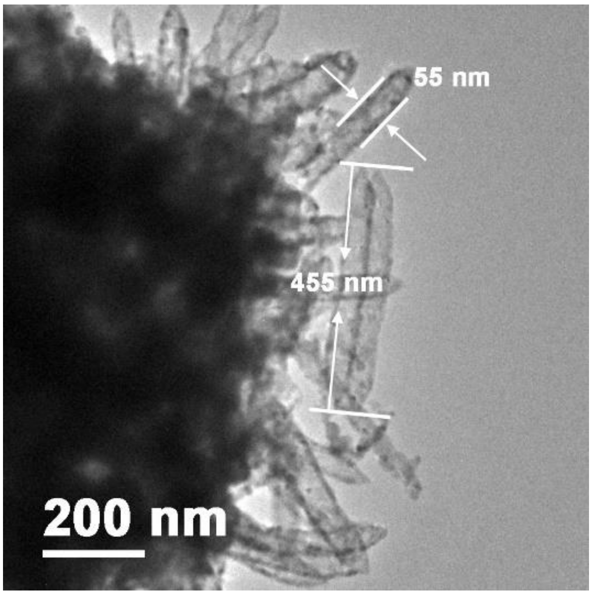

Fig. S1 Magnified TEM image of the Fe_2O_3 nanotubes@Co_3O_4 composites

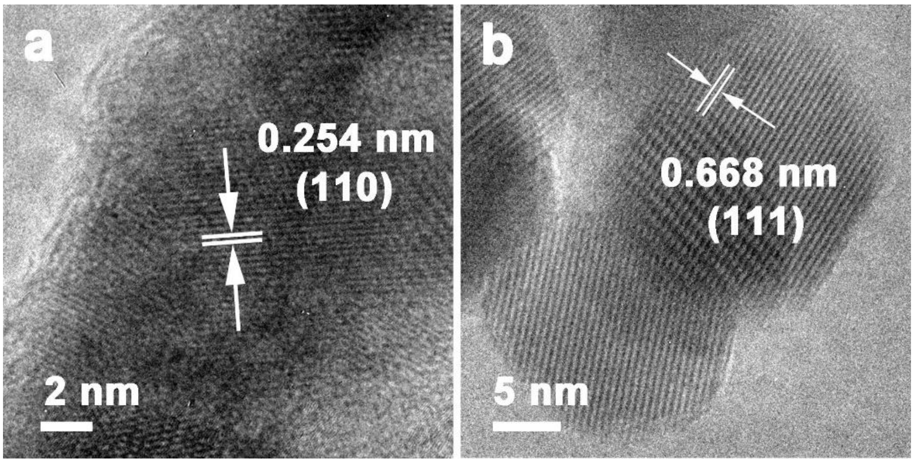

Fig. S2 HRTEM images of **a** Fe_2O_3 nanotube and **b** Co_3O_4 host

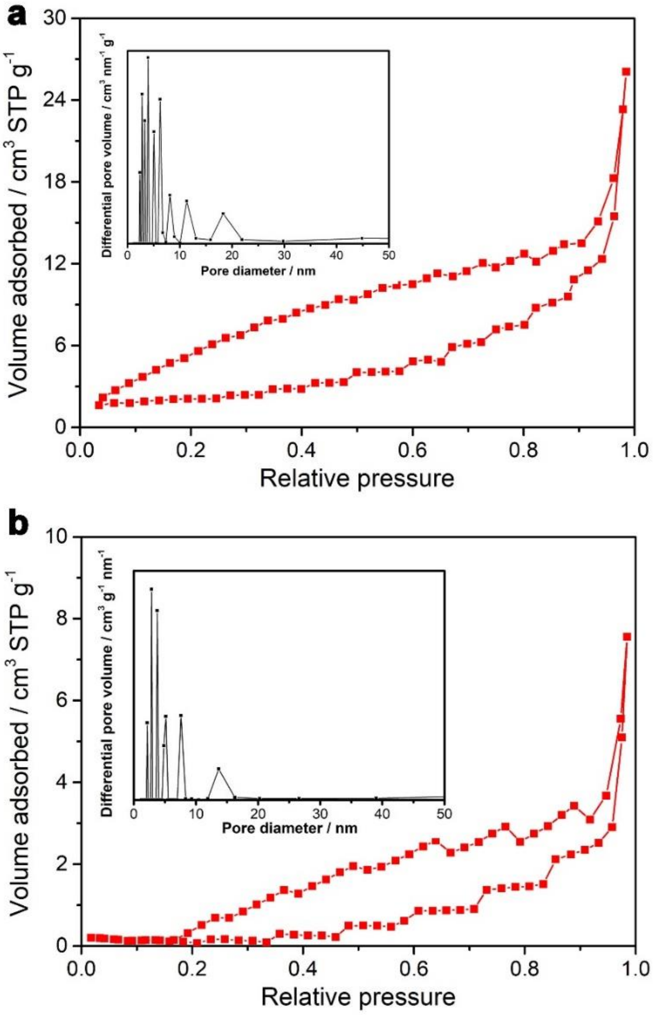

Fig. S3 N_2 sorption isotherms (inset: pore size distributions) of **a** Fe_2O_3 and **b** Co_3O_4 nanostructures derived from MIL-88B and ZIF-67, respectively

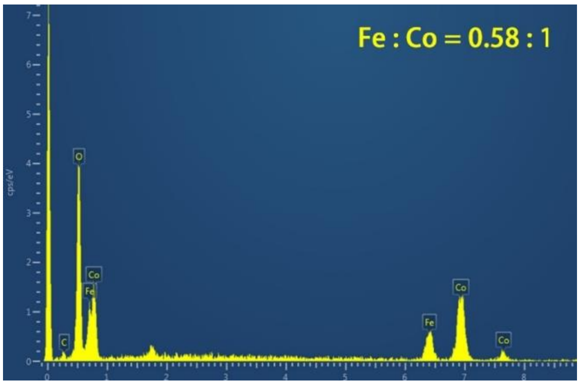

Fig. S4 EDX spectrum of Fe_2O_3 nanotubes@Co_3O_4 composites

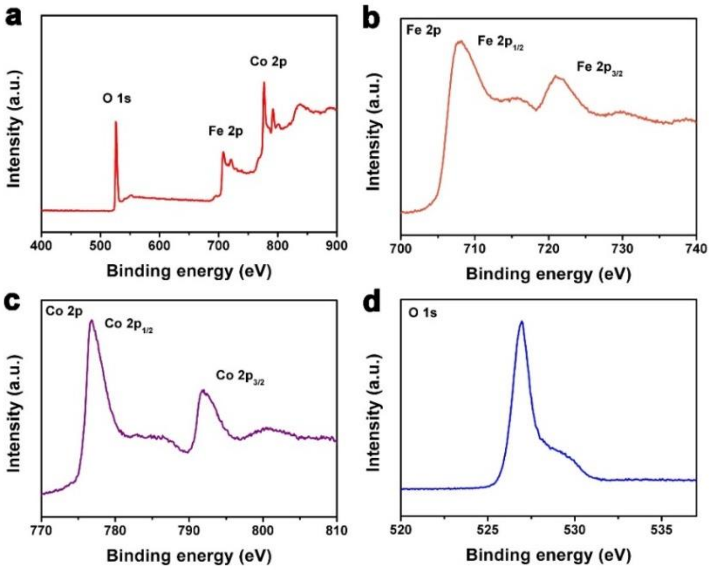

Fig. S5 XPS spectra of Fe_2O_3 nanotubes@Co_3O_4 composites: **a** survey spectrum, and high-resolution spectra of **b** Fe 2p, **c** Co 2p, and **d** O 1s

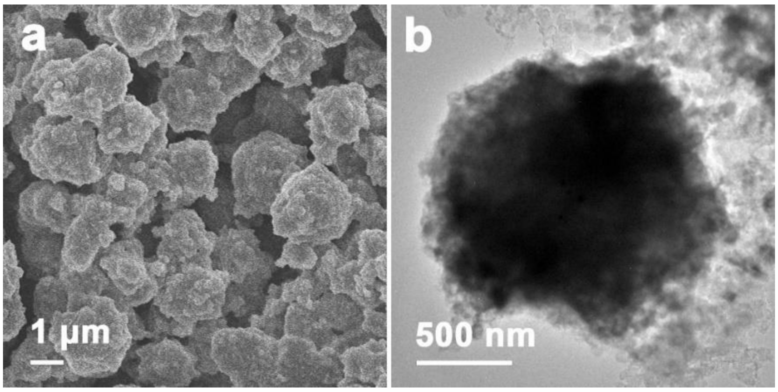

Fig. S6 **a** FESEM and **b** TEM images of the Fe_2O_3 nanotubes@Co_3O_4 composites after cycling for 80 cycles

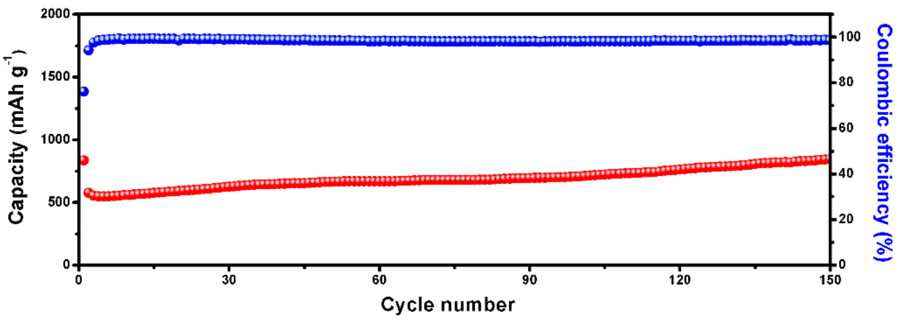

Fig. S7 The cycling performance of Fe_2O_3 nanotubes@Co_3O_4 composites and corresponding Coulombic efficiency at the current density of 1.0 A g^{-1}

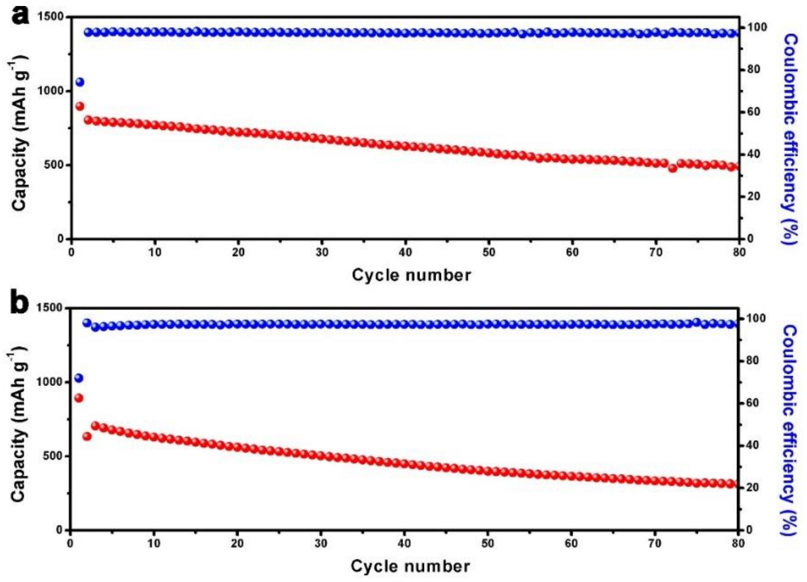

Fig. S8 Cycling performance of MIL-88B and ZIF-67 derived **a** Fe_2O_3 and **b** Co_3O_4 nanostructures and corresponding Coulombic efficiency at the current density of 0.5 A g^{-1}

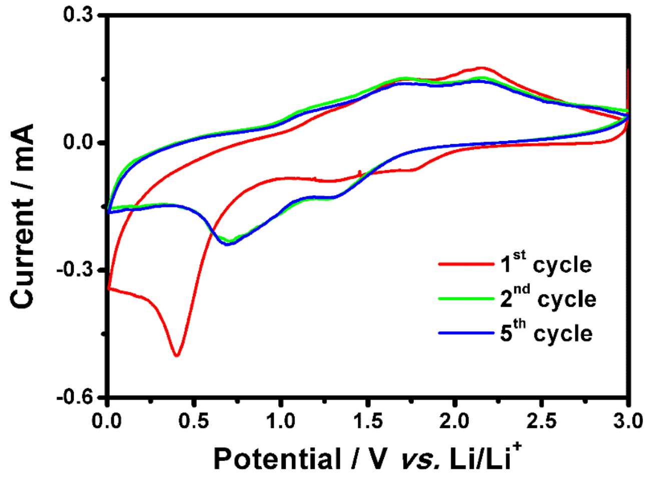

Fig. S9 CV curves of Fe_2O_3 nanotubes@Co_3O_4 composites

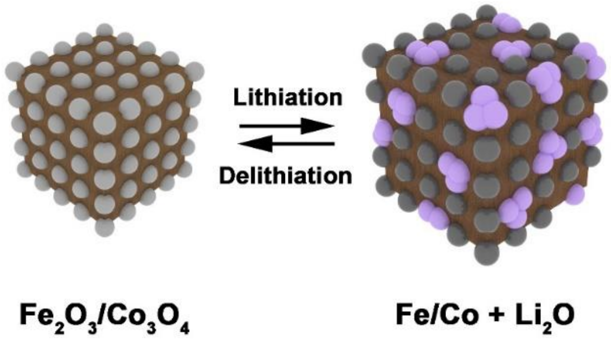

Fig. S10 A schematic representation of the conversion reaction mechanism in Fe_2O_3/Co_3O_4 electrode materials for lithium ion batteries

Table S1 Electrochemical performance of different Fe_2O_3, Co_3O_4, and their composite electrodes

Type of materials	Capacity (mAh g^{-1})	Rate performance	Loading mass	Reference
Fe_2O_3@carbon aerogel composite	725.6 (0.1 A g^{-1})	70.6% from 0.1 to 1 A g^{-1}	NA	[1]
Fe_2O_3-filled CNTs	565 (0.06 A g^{-1})	59.2% from 0.06 to 1.2 A g^{-1}	NA	[2]
Fe_2O_3 nanorods	896 (0.2 A g^{-1})	42.9% from 0.2 to 2.4 A g^{-1}	NA	[3]
carbon/Co_3O_4 nanospheres	738 (0.05 A g^{-1})	57.3% from 0.05 to 2 A g^{-1}	NA	[4]
Co_3O_4/C nanowires	842.3 (0.5 A g^{-1})	26.1% from 0.5 to 8 A g^{-1}	NA	[5]
Co_3O_4 double-shelled hollow spheres	866 (0.178 A g^{-1})	57.8% from 0.178 to 1.78 A g^{-1}	NA	[6]
hybrid Co_3O_4– Fe_2O_3/C particles	782 (0.0731 A g^{-1})	50.5% from 0.0731 to 2.924 A g^{-1}	1.5-2.0 mg cm^{-2}	[7]
Co_3O_4/Fe_2O_3 branched nanowires	980 (0.1 A g^{-1})	NA	1.0-2.0 mg cm^{-2}	[8]
Fe_2O_3 nanotubes@Co_3O_4 composites	726.2 (0.1 A g^{-1})	81.3% from 0.1 to 2 A g^{-1}	0.5-0.8 mg cm^{-2}	This work

References

[1] D. Luo, F. Lin, W. Xiao, W. Zhu, Synthesis and electrochemical performance of α-Fe_2O_3@carbon aerogel composite as an anode material for Li-ion batteries. Ceram. Int. **43**, 2051-2056 (2017). https://doi.org/10.1016/j.ceramint.2016.10.178

[2] W. Yu, P. Hou, L. Zhang, F. Li, C. Liu, H. Cheng, Preparation and electrochemical property of Fe_2O_3 nanoparticles-filled carbon nanotubes. Chem. Commun. **46**, 8576-8578 (2010). https://doi.org/10.1039/c0cc02121k

[3] M. Chen, E. Zhao, Q. Yan, Z. Hu, X. Xiao, D. Chen, The effect of crystal face of Fe_2O_3 on the electrochemical performance for lithium-ion batteries. Sci. Rep. **6**, 29381 (2016). https://doi.org/10.1038/srep29381

[4] N. Jayaprakash, W.D. Jones, S.S. Moganty, L.A. Archer, Composite lithium battery anodes based on carbon@Co_3O_4 nanostructures: Synthesis and characterization. J. Power Sources **200**, 53-58 (2012). https://doi.org/10.1016/j.jpowsour.2011.10.018

[5] P. Zhang, Z. Guo, Y. Huang, D. Jia, H. Liu, Synthesis of Co_3O_4/Carbon composite nanowires and their electrochemical properties. J. Power Sources **196**, 6987-6991 (2011). https://doi.org/10.1016/j.jpowsour.2010.10.090

[6] X. Wang, X. Wu, Y. Guo, Y. Zhong, X. Cao, Y. Ma, J. Yao, Synthesis and lithium storage properties of Co_3O_4 nanosheet-assembled multishelled hollow spheres. Adv. Funct. Mater. **20**, 1680-1686 (2010). https://doi.org/10.1002/adfm.200902295

[7] I. Sultana, M. Rahman, T. Ramireddy, N. Sharma, D. Poddar, A. Khalid, H. Zhang, Y. Chen, A.M. Glushenkov, Understanding structure–function relationship in hybrid Co_3O_4–Fe_2O_3/C lithium-ion battery electrodes. ACS Appl. Mater. Interfaces **7**, 20736-20744 (2015). https://doi.org/10.1021/acsami.5b05658

[8] H. Wu, M. Xu, Y. Wang, G. Zheng, Branched Co_3O_4/Fe_2O_3 nanowires as high capacity lithium-ion battery anodes. Nano Res. **6**, 167-173 (2013). https://doi.org/10.1007/s12274-013-0292-z
